# *SPX1* is an important component in the phosphorus signalling network of common bean regulating root growth and phosphorus homeostasis

**DOI:** 10.1093/jxb/eru183

**Published:** 2014-04-30

**Authors:** Zhu-Fang Yao, Cui-Yue Liang, Qing Zhang, Zhi-Jian Chen, Bi-Xian Xiao, Jiang Tian, Hong Liao

**Affiliations:** State Key Laboratory for Conservation and Utilization of Subtropical Agro-bioresources, Root Biology Center, South China Agricultural University, Guangzhou 510642, PR China

**Keywords:** Bean, hairy roots, phosphate starvation, phosphorus homeostasis, root growth, SPX domain.

## Abstract

Proteins containing the SPX domain are believed to play vital roles in the phosphorus (P) signalling network in plants. However, the functions of SPX proteins in legumes remain largely unknown. In this study, three SPX members, *PvSPX1–PvSPX3* were cloned from common bean (*Phaseolus vulgaris* L.). It was found that the transcripts of all three *PvSPX* members were significantly enhanced in both bean leaves and roots by phosphate (Pi) starvation. Among them, the expression of nuclear localized *PvSPX1* showed more sensitive and rapid responses to Pi starvation. Consistently, only overexpression of *PvSPX1* resulted in increased root P concentration and modified morphology of transgenic bean hairy roots, such as inhibited root growth and an enlarged root hair zone. It was further demonstrated that *PvSPX1* transcripts were up-regulated by overexpressing *PvPHR1*, and overexpressing *PvSPX1* led to increased transcripts of 10 Pi starvation-responsive genes in transgenic bean hairy roots. Taken together, it is suggested that PvSPX1 is a positive regulator in the P signalling network of common bean, and is downstream of PvPHR1.

## Summary

PvSPX1 was found to be a positive regulator in the P signalling network of common bean, and is downstream of PvPHR1.

## Introduction

Phosphorus (P) is an essential element for plant growth, and is easily fixed by soil particles due to its chemical properties. Therefore, low P availability adversely affects crop growth and production, especially on acid soils (Raghothama, 1999; Vance *et al.*, 2003). To cope with low P stress, plants have developed a wide range of adaptive strategies, such as changes in root architecture and morphology (Liao *et al.*, 2004; Zhou *et al.*, 2008; Péret *et al.*, 2011; Tian *et al.*, 2012), increased exudation of protons and organic acids (Fox and Comerford, 1990; Ström *et al.*, 2005; Taghipour and Jalali, 2012), and enhanced secreted or root-associated acid phosphatase activities (Del Pozo *et al.*, 1999; Bozzo *et al.*, 2002; Ligaba *et al.*, 2004; C. Wang *et al.*, 2009; Liang *et al.*, 2010; Robinson *et al.*, 2012; L.S. Wang *et al.*, 2012). These adaptive strategies are tightly mediated by the P signalling network, which is composed of a wide array of regulators (Raghothama, 1999; Vance *et al.*, 2003; Chiou and Lin, 2011).

Proteins containing the SPX domains have been demonstrated to play vital roles in the P signalling networks of yeast (*Saccharomyces cerevisiae*), *Arabidopsis* (*Arabidopsis thaliana*), rice (*Oryza sativa*), and rape (*Brassica napus*) (Ligaba *et al.*, 2004; Duan *et al.*, 2008; C. Wang *et al.*, 2009, 2012; Z. Wang *et al.*, 2009; Liu *et al.*, 2010; Secco *et al.*, 2012a, b; Yang *et al.*, 2012). The SPX domain is named after SYG1/Pho81/XPR1 proteins, which contain a conserved domain in the N-terminal peptides of yeast SYG1 and PHO81, and human XPR1 proteins (Spain *et al.*, 1995; Lenburg and O’Shea, 1996; Battini *et al.*, 1999; Wang *et al.*, 2004).

In yeast, several SPX domain-containing proteins involved in P acquisition and the signalling pathway have been identified (Secco *et al.*, 2012b). PHO81 is a cyclin-dependent kinase (CDK) inhibitor (Lenburg and O’Shea, 1996). Under phosphate (Pi) starvation conditions, PHO81 inhibits the kinase activity of the PHO80–PHO85 complex against the Pho4 transcription factor, which subsequently regulates transcripts of several Pi starvation-responsive genes (Lenburg and O’Shea, 1996). Many yeast Pi transporters, such as Pho84, Pho87, Pho89, Pho90, and Pho91, also possess the SPX domain (Secco *et al.*, 2012b). It is interesting that most SPX domain-harbouring proteins, including Vtc2, Vtc3, Vtc4, and Gde1, appear to play key regulatory roles in P homeostasis in yeast (Secco *et al.*, 2012b).

In plants, four groups of proteins were also found to contain the SPX domain. Among them, three groups of proteins have the SPX domain in the N-terminus and other domains in the C-terminus, including an EXS (ERD1, XPR1, and SYG1), a major facility superfamily (MFS), or a RING-type zinc finger domain (Hamburger *et al.*, 2002; Stefanovic *et al.*, 2007; Lin *et al.*, 2010; Secco *et al.*, 2010; Kant *et al.*, 2011; C. Wang *et al.*, 2012). Similar to the functions of proteins containing the SPX domain in yeast, most of these plant members are involved in regulating P homeostasis in plants. Examples include OsSPX-MFS1 in rice (C. Wang *et al.*, 2012), along with AtPHO1:1 and AtNLA in *Arabidopsis* (Stefanovic *et al.*, 2007; Secco *et al.*, 2010; Kant *et al.*, 2011).

Recently, a specific group of proteins only containing the SPX domain have been characterized in plants, such as *Arabidopsis* and rice (Duan *et al.*, 2008; C. Wang *et al.*, 2009; Z. Wang *et al.*, 2009; Liu *et al.*, 2010; Yang *et al*., 2011). In *Arabidopsis*, four members only contain the SPX domain, namely AtSPX1, AtSPX2, AtSPX3, and AtSPX4 (Duan *et al.*, 2008). Furthermore, expression patterns of several Pi starvation-responsive genes were positively and negatively regulated by *AtSPX1* and *AtSPX3*, respectively (Duan *et al.*, 2008). Similarly, the negative regulatory role of *OsSPX1* was also suggested in rice, because transcription of several Pi starvation-responsive genes (e.g. *OsPT2*, *OsPT6*, and *OsPAP10*) was suppressed through *OsSPX1* overexpression (C. Wang *et al.*, 2009). Furthermore, it has recently been determined that *OsSPX1* is downstream of *OsPHR2* and *OsPHO2* in the rice P signalling pathway (Liu *et al.*, 2010).

Despite accumulated knowledge of the P signalling network in model plants (i.e. *Arabidopsis* and rice) (Chiou and Lin, 2011), information on Pi starvation-responsive pathways in other crops remains fragmentary. A group of Pi starvation-responsive genes (e.g. *PvmiR399* and *PvPS2:1*) have been cloned and characterized in common bean (*Phaseolus vulgaris* L.), an important legume crop (Tian *et al.*, 2007; Valdes-Lopez *et al.*, 2008; Hernández *et al.*, 2009; Liang *et al.*, 2012a,b). This has facilitated elucidation of the P signalling network in bean, although this knowledge remains incomplete. Recently, essential roles for *PvPHR1* and *PvmiR399* have been suggested in P deficiency signalling (Valdes-Lopez *et al.*, 2008). Nevertheless, other regulators are probably required as well. In a previous study, three expressed sequence tags (ESTs) with high homology to *AtSPX1* were identified through screening a suppression subtractive hybridization library constructed from P-deficient bean (Tian *et al.*, 2007). Among them, the full-length cDNA of *PvIDS4-1* (i.e. *PvSPX1*) was cloned, and its expression levels were found to be up-regulated by Pi starvation in bean (Tian *et al.*, 2007). However, the functions of *PvSPX1* and other *PvSPX* genes in bean adaptation to P deficiency remain unknown. In this study, the full-length cDNA of the other two *PvSPX* genes (i.e. *PvSPX2* and *PvSPX3*) was cloned. Subsequently, the expression patterns and functions of all three *PvSPX* gene family members as related to P availability were characterized in bean.

## Materials and methods

### Plant material and growth conditions

Seeds of common bean genotype G19833 were surface sterilized for 1min using 10% (v/v) H_2_O_2_ and then germinated in the dark on germination paper moistened with 1/4 strength modified nutrient solution as described previously (Yan *et al.*, 2001). Five days after germination, seedlings were transferred to nutrient solution supplied with 5, 50, 100, or 500 μM KH_2_PO_4_ for a P dosage experiment. After 10 d, young leaves and roots were harvested. For time course experiments, seedlings were pre-treated in 1/4 strength nutrient solution for 7 d and then transplanted to nutrient solution containing 5 μM KH_2_PO_4_. Shoots and roots were each harvested at 0, 4, and 8 d after treatment for determination of fresh weight, total root length, and P content. Young leaves and roots were separately harvested for RNA extraction. Nutrient solution was well aerated and its pH was maintained between 5.8 and 6.0. Four biological replicates were included in all of the experiments.

### Analysis of total root length and P content

Roots were scanned, and then the digital images were analysed using Win-Rhizo software (Régent Instruments, Canada) to measure total root length. Shoots and roots were kept separately at 75 °C until completely dry, and then were ground into powder for total P content analysis. P content was determined using the phosphorus–molybdate blue colour reaction as previously described (Murphy and Riley, 1962)

### Cloning full length cDNAs of *PvSPX2* and *PvSPX3*

Gene-specific primers were designed according to the EST sequences of *PvSPX2* (EG594307) and *PvSPX3* (EG594308) (Tian *et al.*, 2007) (Supplementary Table S1 available at *JXB* online). Using the full-length cDNA library constructed from the roots of G19833 as a template, the 5’ and 3’ termini of each gene were amplified by the specific primers paired with T3 and T7 primers, respectively. The amplified DNA fragments were then cloned into the pMD18-T vector (TaKaRa, Japan) and sequenced. Sequences of *PvSPX2* and *PvSPX3* were analysed at the National Center for Biotechnology Information (NCBI) website (http://www.ncbi.nlm.nih.gov/), and deposited in GenBank with accession numbers GU189405 for *PvSPX2* and GU189406 for *PvSPX3*. Multiple sequence alignments were conducted using ClustalW 1.8. The phylogenetic tree was established using the Neighbor–Joining method of the MEGA 4.1 program.

### RNA extraction and quantitative real-time PCR

Total RNA was isolated from young leaves and roots using RNAiso Plus reagent (TaKaRa) and treated with DNase I (TaKaRa). The first-strand cDNA was synthesized from total RNA using MMLV reverse transcriptase following the manual (Promega Inc., USA). The first-strand cDNA was then used for SYBR Green-monitored quantitative real-time PCR (qPCR) analysis, which was performed using a Rotor-Gene 3000 (Corbett Research, Australia). Expression levels of the tested genes were quantified relative to expression levels of the reference gene *EF-1α* (PvTC3216) using arbitrary units. The primer pairs used for qPCR analysis are shown in supplementary Table S1 at *JXB* online. All of the gene expression analyses had four biological replicates.

### Subcellular localization analysis

The coding regions of *PvSPX1* (EF191350), *PvSPX2*, and *PvSPX3* without stop codons were separately cloned into the transient expression vector (*pBEGFP*), and fused with green fluorescent protein (GFP; Liang *et al.*, 2010). For subcellular localization of PvSPXs in onion (*Allium cepa*) epidermal cells, the *PvSPX–GFP* fusion constructs and *GFP* empty vector control were separately transformed into onion epidermal cells using a helium-driven accelerator (PDS/1000, Bio-Rad). After the transformed cells were cultured on Murashige and Skoog (MS) medium for 16h, the GFP florescence was observed using a confocal scanning microscope system (TCS SP2, Leica, Germany) with 488nm excitation and 500–525nm emission filter wavelengths. For subcellular localization of PvSPXs in leaf epidermal cells of tobacco (*Nicotiana tabacum*), the *PvSPX–GFP* fusion constructs and GFP empty vector control were separately transformed into *Agrobacterium tumefaciens* strain GV3101, which were further used for transformation as previously described (Sparkes *et al.*, 2006). After the transformation, plants were grown under normal conditions for 48h and the GFP florescence was observed using a fluorescence microscope (Leica DM5000B). The GFP fluorescence was imaged using a Leica DFC 480 camera.

### Transformed genes in common bean hairy roots

The coding regions of *PvSPX1*, *PvSPX2*, *PvSPX3*, and *PvPHR1* (EU500763) were inserted separately into the unique *Bam*HΙ and *Mlu*Ι sites of the binary vector pYLRNAi as previously described (Liang *et al.*, 2010). For *PvSPX1* RNA interference (RNAi) construction, the same binary vector pYLRNAi was used by inserting the *PvPSX1*-specific fragment into the *Bam*HΙ and *Hin*dIII, and the *Pst*I and *Mlu*I sites, respectively. The overexpression, RNAi constructs, and the empty vector control (CK) were then separately transformed into *Agrobacterium rhizogenes* strain K599, which were further used for hairy root transformation. Transformed bean hairy roots were generated and maintained as described previously (Liang *et al.*, 2012b). Briefly, sterilized bean seeds were germinated on half-strength MS medium. After 35h, the abaxial sides of cotyledons were wounded with a scalpel previously dipped into the overnight cultures of the transgenic *A. rhizogenes* strain K599. The wounded cotyledons were cultured in solid MS medium to develop hairy roots. The expression levels of the corresponding genes in hairy roots were verified through qPCR analysis. For P treatments, ~0.2g (fresh weight) of hairy roots was cultured in solid MS medium with or without the addition of 1.25mM KH_2_PO_4_. After 14 d growth, transgenic bean hairy roots were photographed using a microscope (Leica) and a Leica DFC 480 camera. The fresh weight and total P content of each transgenic line were determined as described above. The lateral root length was analysed using Win-Rhizo. Based on root hair density, lateral roots were separated into two zones, namely the root hair zone (i.e. the part of the root zone with >10 root hairs per 1mm root) and the non-root hair zone, and then the percentage of the root hair zones in the lateral roots was calculated. In total, 10 lateral roots were analysed for each replicate. For each treatment, four biological replicates were included.

To analyse the expression patterns of genes downstream of *PvSPX1* in P signalling, total RNA was extracted from transgenic hairy roots grown under high P conditions. Subsequently, qPCR was conducted to analyse the expression of 11 genes downstream of *PvSPX1*: *PvPT1* (TC27368), *PvPHT2* (TC30856), *Pv4* (CV536419), *PvPAP1* (BAD05166), *PvPAP2* (CAA04644), *PvPAP3* (AC025293), *PvPAP4* (AAF60317), *PvPAP5* (ADK56125), *PvPS2:1* (EF472460), *PvLPR1-like* (FE710903), and *PvPDR2-like* (TC44308). All qPCR primers (Supplementary Table S1 at *JXB* online) were designed according to the sequences downloaded from the Dana-Farber Cancer Institute (DFCI; http://compbio.dfci.harvard.edu/tgi/) for *PvPHT2*, *PvLPR1-like*, and *PvPDR2-like*, or from the GenBank database (http://www.ncbi.nlm.nih.gov/genbank) entries for *PvPHR1*, *PvPT1*, *Pv4*, *PvPAP1*, *PvPAP2*, *PvPAP3*, *PvPAP4*, *PvPAP5*, and *PvPS2:1*. To construct the *PvSPX1* and *PvSPX2* promoter fused with β-glucuronidase (*GUS*) vectors, the 5’-regulatory regions of 1.9kb for *PvSPX1* and 1.6kb for *PvSPX2* were each cloned, and inserted into the pCAMBIA 1391 vector. The constructs were transformed into the bean hairy roots as described above, and GUS activity was analysed as described before (Liang *et al.*, 2012a).

## Results

### Plant growth was affected by P availability

P deficiency significantly inhibited bean growth, as reflected by decreases in plant fresh weight, total root length, and total P content (Supplementary Table S2 at *JXB* online). With an increased duration of P deficiency, plant fresh weight and total P content gradually decreased. After 8 d of Pi starvation, the total P contents of bean shoots and roots were reduced by 32% and 30%, respectively, as compared with high P conditions (Supplementary Table S2). Similarly, total root length was also significantly decreased by P deficiency. Total root length at 4 d and 8 d of P deficiency was reduced by 39% and 55%, respectively, as compared with under high P conditions (Supplementary Table S2).

### Identification and bioinformatics analysis of *PvSPX2* and *PvSPX3*

Based on the reported EST sequences of *PvSPX2* and *PvSPX3*, the full-length cDNAs of both *PvSPX2* and *PvSPX3* were cloned from a full-length cDNA library of G19833 subjected to P deficiency. The coding regions of *PvSPX2* and *PvSPX3* were 861bp and 756bp in length, respectively. Alignment analysis showed that PvSPX2 and PvSPX3 exhibited 75% and 50% similarity to PvSPX1, respectively.

**Fig. 1.**
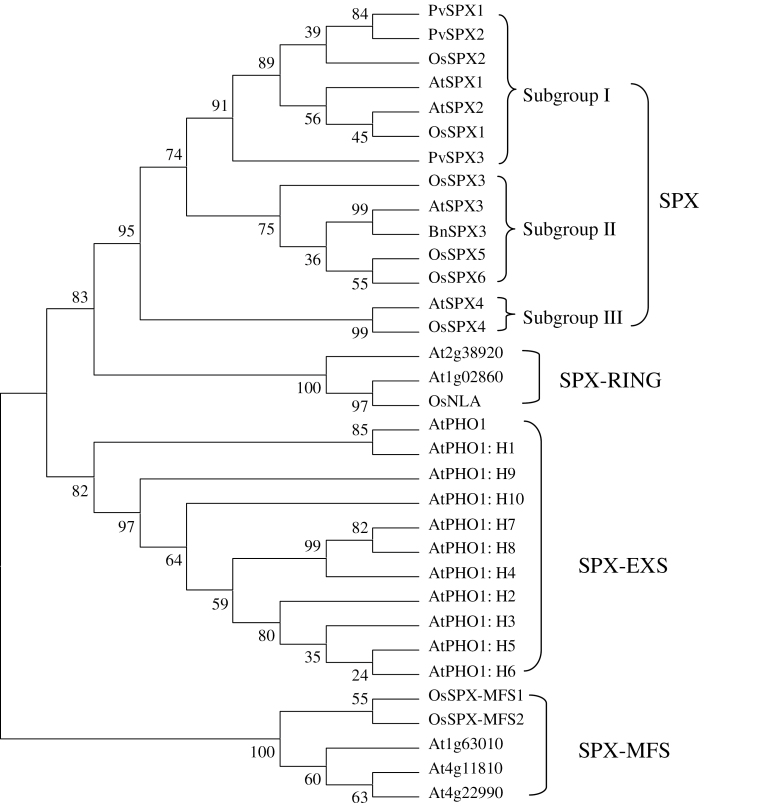
Phylogenetic analysis of SPX proteins in plants. The first two letters of each protein label represent the abbreviated species name: At, *Arabidopsis thaliana*; Os, *Oryza sativa*; Pv, *Phaseolus vulgaris*. AtSPX1 (At5g20150), AtSPX2 (At2g26660), AtSPX3 (At2g45130), AtSPX4 (At5g15330), AtPHO1 (AT3G23430), AtPHO1: H1 (At1g68740), AtPHO1: H2 (At2g03260), AtPHO1: H3 (At1g14040), AtPHO1: H4 (At4g25350), AtPHO1: H5 (At2g03240), AtPHO1: H6 (At2g03250), AtPHO1: H7 (At1g26730), AtPHO1: H8 (At1g35350), AtPHO1: H9 (At3g29060), AtPHO1: H10 (At1g69480), OsSPX1 (Os03g0343400), OsSPX2 (Os02g10780), OsSPX3 (Os10g25310), OsSPX4 (Os03g61200), OsSPX5 (Os03g29250), OsSPX6 (Os07g42330), OsNLA (Os02g0673200), OsSPX-MFS1 (Os04g0573000), OsSPX-MFS2 (Os02g0678200), PvSPX1 (EF191350), PvSPX2 (EG594307), PvSPX3 (EG594308).

Phylogenetic analysis showed that plant proteins containing the SPX domain could be divided into four groups, namely SPX, SPX-EXS, SPX-MFS, and SPX-RING (Fig. 1). Furthermore, SPX proteins could be further subdivided into three groups. Among them, PvSPX1, PvSPX2, and PvSPX3 belong to group I, which includes AtSPX1 and AtSPX2 in *Arabidopsis*, as well as OsSPX1 and OsSPX2 in rice (Fig. 1).

### Temporal expression patterns of *PvSPX* genes in response to Pi starvation

**Fig. 2.**
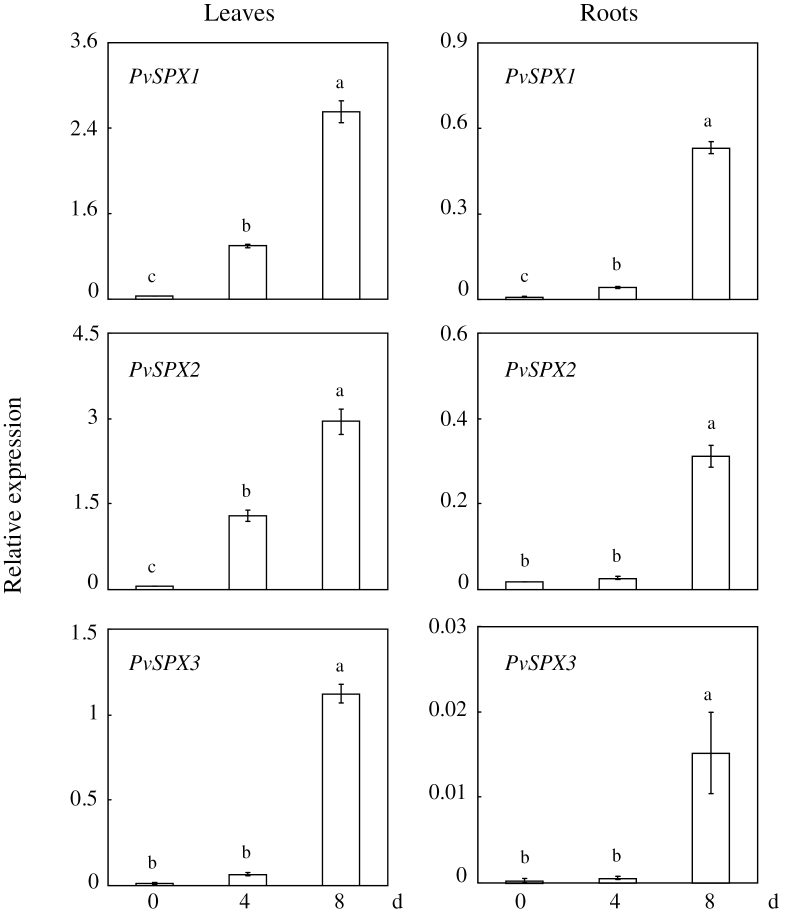
Temporal expression patterns of *PvSPX* genes in response to Pi starvation in leaves and roots of bean. Seedlings were hydroponically grown under normal conditions for 7 d, and then subjected to P deficiency for 0, 4, and 8 d. Total RNA isolated from leaves and roots of plants was used for qPCR analysis. Expression levels of the tested genes were quantified relative to expression levels of the reference gene *EF-1α* (PvTC3216) using arbitrary units. Each bar is the mean of four replicates with the standard error. Different letters represent significant differences at the 0.05 level.

The temporal expression patterns of the three *PvSPX* genes in bean leaves and roots were analysed by qPCR. As shown in Fig. 2, their expression levels were significantly increased over time and reached their highest levels after 8 d of low P treatment (Fig. 2). However, their expression patterns varied in leaves and roots at 4 d of P deficiency (Fig. 2). After 4 d of P deficiency, significantly increased transcription was observed for *PvSPX1* and *PvSPX2* in leaves, while for *PvSPX3* transcription was not increased either in leaves or in roots (Fig. 2). This suggests that *PvSPX1* and *PvSPX2* respond to Pi starvation earlier than *PvSPX3* in bean.

### Dosage responses of *PvSPX* genes to P availability

**Fig. 3.**
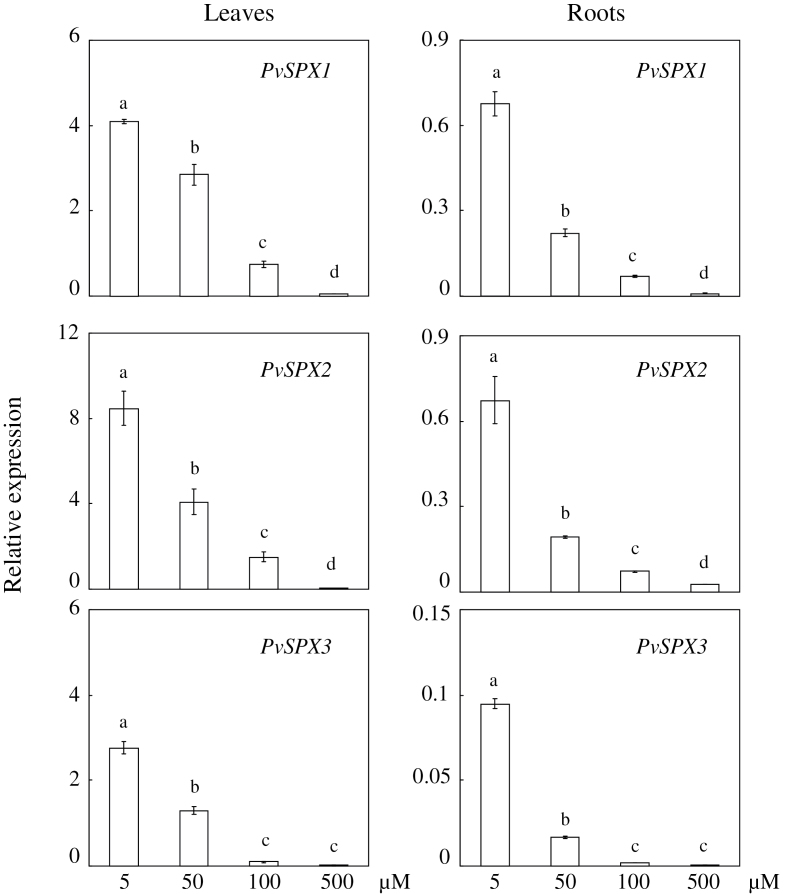
Dosage response of *PvSPX* genes to P deficiency. Seedlings were grown in nutrient solution supplied with 5, 50, 100, or 500 μM KH_2_PO_4_. After 10 d, total RNA was isolated from leaves and roots for qPCR analysis. Expression levels of the tested genes were quantified relative to expression levels of the reference gene *EF-1α* (PvTC3216) using arbitrary units. Each bar is the mean of four replicates with the standard error. Different letters represent significant differences at the 0.05 level.

Expression patterns of the three *PvSPX* members studied here were tightly dependent on P availability in the medium (Fig. 3). Their highest transcript levels were observed in both leaves and roots supplied with 5 μM P, and were decreased with increased P availability (Fig. 3). When the applied P concentration was increased to 500 μM, transcription of each *PvSPX* gene was negligible (Fig. 3). However, slight differences existed among their expression patterns as related to P availability. Transcript levels of *PvSPX1* and *PvSPX2* in both leaves and roots declined significantly when the applied P concentration was increased from 100 μM to 500 μM, but that of *PvSPX3* did not (Fig. 3), suggesting that expression of *PvSPX1* and *PvSPX2* might be more sensitive to P availability than that of *PvSPX3*.

### Subcellular localization of PvSPX proteins

**Fig. 4.**
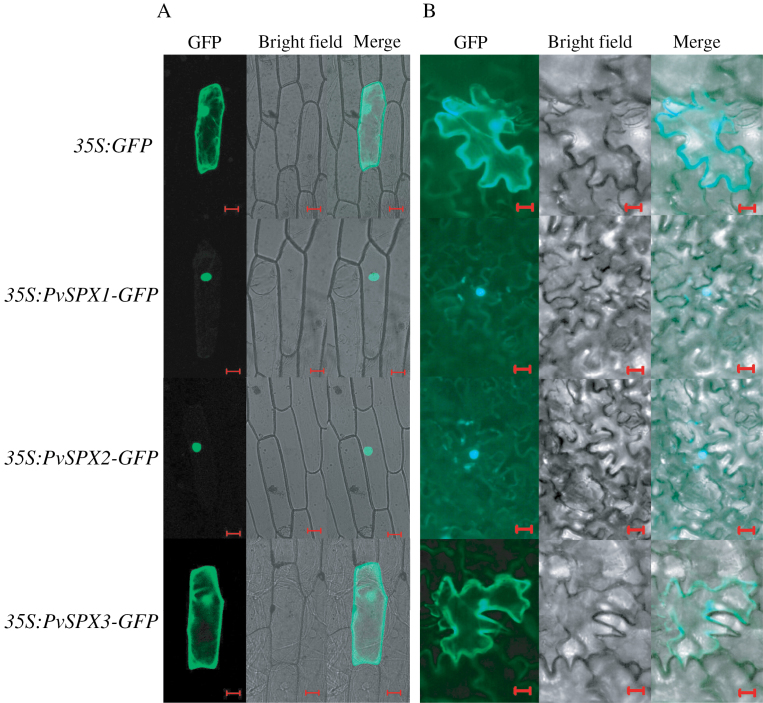
Subcellular localization of PvSPXs. (A) Transient expression of the *pBEGFP* construct and *PvSPX–GFP* fusion in onion epidermal cells. Scale bars=50 μm. (B) Transient expression of the *pBEGFP* construct and *PvSPX–GFP* fusion in tobacco epidermal cells. Scale bars=20 μm. The first row shows the empty vector control, followed by *PvSPX1–GFP*, *PvSPX2–GFP*, and *PvSPX3–GFP* constructs. Cells were observed by the green fluorescence of GFP and the PvSPX–GFP proteins.

To determine the subcellular localization, the coding regions of the three *PvSPX* genes were fused with the *GFP* reporter gene and transiently expressed in onion and tobacco epidermal cells. Subcellular localization was visualized by detecting GFP signal in the transformed onion and tobacco epidermal cells. The empty vector containing *35S:GFP* was used as a control. The results showed that the three PvSPX members were found in various subcellular localizations (Fig. 4). Signals of GFP fusion with PvSPX1 and PvSPX2 were only detected in the nuclei of onion and tobacco epidermal cells (Fig. 4). However, GFP fusion with PvSPX3 was observed in many areas in onion and tobacco epidermal cells, suggesting that PvSPX3 might be localized in the cytoplasm and nuclei (Fig. 4).

### Functional analysis of *PvSPX* genes in transgenic hairy roots

**Fig. 5.**
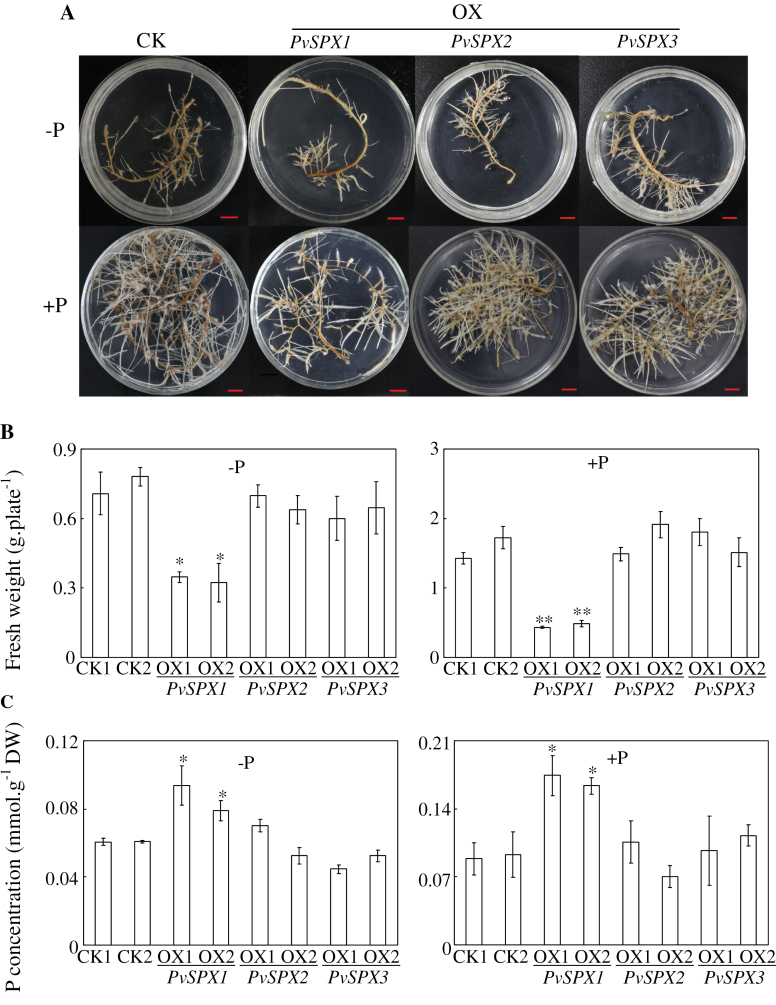
Growth and P concentration of bean hairy roots at two P levels. (A) Photograph of bean hairy roots grown at two P levels. Scale bars=1cm. (B) Fresh weight of bean hairy roots at two P levels. (C) P concentration in bean hairy roots. Bean hairy roots were grown in media containing 0 μM (–P) or 1.25mM (+P) KH_2_PO_4_ for 14 d. THe fresh weight and P concentration were measured. Each bar is the mean of four replicates with the standard error. Asterisks represent significant differences between overexpressing *PvSPX* and CK for the same trait in *t*-tests. *0.01<*P*≤0.05; **P≤0.01. OX1 and OX2 indicate two transgenic bean hairy root lines overexpressing *PvSPX1*, *PvSPX2*, or *PvSPX3*. CK1 and CK2 indicate the two transgenic lines transformed with the empty vector.

The functions of *PvSPX* genes were further analysed in bean transgenic hairy roots by overexpressing *PvSPX1*, *PvSPX2*, and *PvSPX3*. Significantly increased transcripts of the three *PvSPX* genes in the transgenic bean hairy roots were verified through qPCR analysis (Supplementary Fig. S1 at *JXB* online). Subsequently, the transgenic hairy roots were grown in MS medium with or without P application for 14 d. The results showed that only overexpressing *PvSPX1* could inhibit hairy root growth, as reflected by reduced fresh weight of hairy roots under both P conditions (Fig. 5A, B). Compared with the control lines, the fresh weight of the *PvSPX1* overexpression line was reduced by ~60% in high P and 40% in low P (Fig. 5B). Furthermore, the P concentration in the *PvSPX1* overexpression line was higher than that in the control line by ~45% in high P and 30% in low P (Fig. 5C). In contrast, the fresh weight and P concentration of both *PvSPX2* and *PvSPX3* overexpression lines were similar to those in the control line at the two P levels (Fig. 5B, C). Similarly, suppressed *PvSPX1* did not affect hairy root fresh weight and P concentration, compared with those in the empty vector (CK) controls (Supplementary Figs S2, S3).

**Fig. 6.**
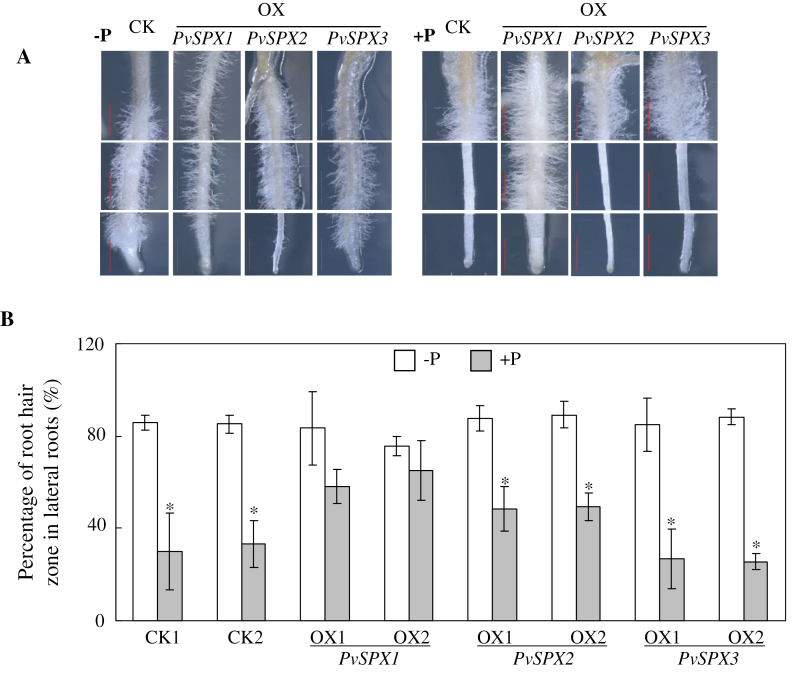
Root morphology and the percentage of the root hair zones in bean hairy roots at two P levels. (A) Photographs of roots in bean hairy roots at two P levels. (B) Percentage of the root hair zone in bean hairy roots at two P levels. Bean hairy roots were grown in media containing 0 μM (–P) or 1.25mM (+P) KH_2_PO_4_ for 14 d. Ten lateral roots were selected from each replicate for further analysis. Photographs were taken of three zones of the lateral roots, namely the root tip, root middle, and root base. Scale bars=1mm. OX1 and OX2 indicate two transgenic bean hairy root lines overexpressing *PvSPX1*, *PvSPX2*, or *PvSPX3*. CK1 and CK2 indicate the two transgenic lines transformed with the empty vector. Asterisks represent significant differences between two P treatments for the same trait at the 0.05 level.

Root morphology was further investigated in all hairy root lines at the two P levels through determination of the percentage of the root hair zones (i.e. the part of root zone with >10 root hairs per 1mm root) in bean hairy roots. The percentage of the root hair zones in all hairy root lines was ~80% without P application (Fig. 6). With P application, the percentage of the root hair zones of CK, and *PvSPX2* and *PvSPX3* overexpression lines was decreased by >50% (Fig. 6). However, for the *PvSPX1* overexpression line, applied P did not affect the percentage of the root hair zone (Fig. 6). Interestingly, similar results were also observed in *PvSPX1* RNAi lines, in which the percentage of the root hair zone was not affected by P application (Supplementary Fig. S4 at *JXB* online). The results suggest that expression of *PvSPX1* might regulate enlargement of the root hair zones at a high P level.

### PvSPX participates in the P signalling network in bean

**Fig. 7.**
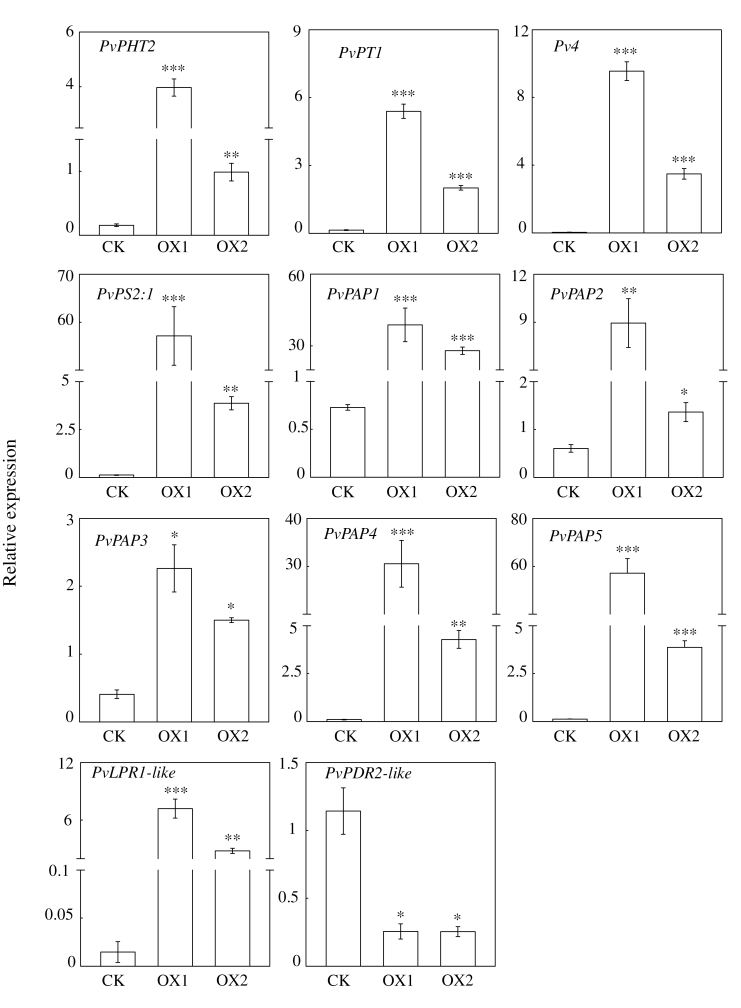
Transcription levels of downstream genes of *PvSPX1* in CK and *PvSPX1*-overexpressing bean hairy roots. Expression patterns of downstream genes were determined by qPCR in CK and two *PvSPX1* overexpression hairy root lines grown in MS medium containing 1.25mM P. Expression levels of the tested genes were quantified relative to expression levels of the reference gene *EF-1α* (PvTC3216) using arbitrary units. OX1 and OX2 indicate two transgenic bean hairy root lines overexpressing *PvSPX1*. CK indicates the transgenic line transformed with the empty vector. Asterisks represent significant differences of downstream gene expression levels between *PvSPX1*-overexpressing and CK in *t*-tests. *0.01<*P*≤0.05; **0.001<*P*≤0.01; ***P≤0.001.

**Fig. 8.**
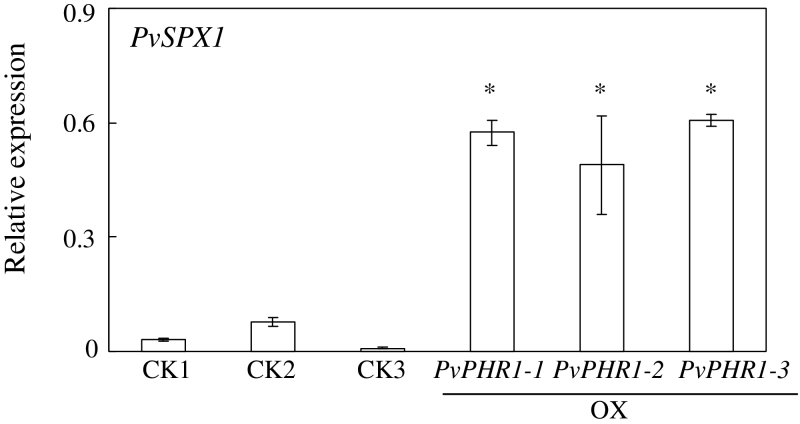
*PvSPX1* transcripts in CK and *PvPHR1*-overexpressing transgenic bean hairy roots. Expression levels of *PvSPX1* were determined in CK and three *PvPHR1*-overexpressing hairy root lines grown in MS medium containing 1.25mM P by qPCR. Expression levels of the tested genes were quantified relative to the expression levels of the reference gene *EF-1α* (PvTC3216) using arbitrary units. *PvPHR1-1*, *PvPHR1-2*, and *PvPHR1-3* indicate three transgenic bean hairy root lines overexpressing *PvPHR1*. CK1, CK2, and CK3 indicate three transgenic lines transformed with the empty vector. An asterisk indicates a significant difference in *PvSPX1* expression between *PvPHR1*-overexpressing and CK lines at the 0.05 level.

The expression patterns of 11 genes were investigated in the transgenic hairy roots overexpressing *PvSPX1* in order to illustrate the regulatory role of *PvSPX1* in the P signalling network in bean. Among them, nine genes were previously characterized as Pi starvation-responsive genes, namely two Pi transporters (*PvPT1* and *PvPHT2*), five purple acid phosphatases (*PvPAP1–PvPAP5*), *Pv4*, and *PvPS2:1*. The other two genes (*PvLPR1-like* and *PvPDR2-like*) exhibit high homology with *AtLPR1* and *AtPDR2*, respectively, which both regulate root growth in *Arabidopsis*. The qPCR analysis showed that overexpressing *PvSPX1* led to significantly increased transcription of 10 genes compared with the control line, namely *PvPT1*, *PvPHT2*, *Pv4*, *PvPAP1–PvPAP5*, *PvPS2:1*, and *PvLPR1-like* (Fig. 7). Consistently, suppressed transcripts of *PvSPX1* resulted in lower expression patterns of several genes—*PvPHT2*, *PvPAP3*, *PvPS2:1*, and *PvLPR1-like* (Supplementary Fig. S5 at *JXB* online). The results suggest that expression of these genes is positively regulated by *PvSPX1*. However, expression levels of *PvPDR2-like* were inhibited in the *PvSPX1* overexpression lines and increased in the *PvSPX1* RNAi lines, compared with those in the control line (Fig. 7; Supplementary Fig. S7), suggesting that *PvPDR2-like* is negatively regulated by *PvSPX1* in bean. Similarly, *PvSPX2* overexpression resulted in increased transcripts of several genes downstream of *PvSPX1*, except *PvPDR2-like* (Supplementary Fig. S6), suggesting that PvSPX2 might have a similar regulatory role to PvSPX1. However, overexpression of *PvSPX3* did not affect expression patterns of genes downstream of *PvSPX1* (Supplementary Fig. S6). Furthermore, significantly increased transcription of *PvSPX1* was obviously observed in the transgenic bean hairy roots with overexpression of *PvPHR1* (Fig. 8), suggesting that *PvSPX1* lies downstream of *PvPHR1*.

## Discussion

Proteins containing the SPX domain have been well documented to be involved in the P signalling pathway of yeast and model plants, including *Arabidopsis* and rice (Lenburg and O’Shea, 1996; Duan *et al.*, 2008; C. Wang *et al.*, 2009; Z. Wang *et al.*, 2009; Lin *et al.*, 2010; Secco *et al.*, 2012a). However, involvement of SPX proteins in P signalling remains largely unknown in legumes. In this study, three *PvSPX* genes were cloned and comparatively characterized as related to Pi starvation in bean. The results demonstrated that *PvSPX1* is an important regulator in the P signalling network of common bean, which shows several novel functions in regulating root growth, P homeostasis, and downstream gene transcription.

Since the transcription of several Pi starvation-responsive genes was noticeably increased and decreased in the *PvSPX1* overexpression and RNAi transgenic bean hairy roots, respectively, PvSPX1 appears to be a positive regulator in the bean P signalling network (Fig. 7; Supplementary Fig. S7 at *JXB* online). Furthermore, *PvSPX1* appears to be a downstream gene of *PvPHR1*, because overexpressing *PvPHR1* led to increased transcription of *PvSPX1* in bean hairy roots (Fig. 8). Similarly, it has been demonstrated that *AtSPX1* and *OsSPX1* were downstream genes of *AtPHR1* and *OsPHR2* in the P signalling pathways of *Arabidopsis* and rice, respectively (Duan *et al.*, 2008; C. Wang *et al.*, 2009; Liu *et al.*, 2010). However, regulatory roles of *PvSPX1*, *AtSPX1*, and *OsSPX1* in the P signalling pathways seemed to vary among species despite them showing several similar properties, such as nuclear localization and Pi starvation-induced expression patterns (Figs 2–4). In rice, OsSPX1 has been considered as a negative regulator in the P signalling network because overexpressing *OsSPX1* significantly suppressed the expression levels of 10 Pi starvation-induced genes (C. Wang *et al.*, 2009). Also, *OsSPX1* suppression resulted in increased transcripts of *OsPT2* and *OsPT8* in rice (C. Wang *et al.*, 2009; Liu *et al.*, 2010). However, AtSPX1 was considered as a positive regulator in the *Arabidopsis* P signalling pathway because overexpressing *AtSPX1* led to increased transcription of several genes increased by Pi starvation, such as *AtACP5* and *AtRNS1* (Duan *et al.*, 2008). Therefore, it seems that the regulatory roles of *SPX1* in dicots might differ from those in monocot plants, which needs to be further studied.

Consistent with the enhanced expression levels of two Pi transporter genes (*PvPHT2* and *PvPT1*), a significantly increased P concentration was observed in bean hairy roots overexpressing *PvSPX1*, especially under high P conditions (Fig. 5C). This suggests that *PvSPX1* is involved in regulating P homeostasis in bean roots. Similarly, it has been documented that suppressed expression of *OsSPX1* led to more P accumulation in both leaves and roots in rice under high P conditions (C. Wang *et al.*, 2009; Liu *et al.*, 2010). Taken together, these results suggest that *SPX* might control P homeostasis in plants through regulating expression of Pi transporter (*PT*) genes. However, the molecular mechanisms underlying *SPX* regulation of *PT* transcription remain largely unknown. Since *PvSPX1* has regulatory roles which appear to contrast with those of *OsSPX1* and *AtSPX3* in P signalling pathways, it is plausible that *SPX* might not directly control downstream gene expression. It will be important to clarify the functions of SPX through identification of other P signalling regulators interacting with SPX in plants.

Another novel feature of *PvSPX1* is its involvement in regulating root growth and root morphology in bean roots. Changes in root morphology, such as inhibition of root elongation and stimulation of root hair growth, are well accepted as typical responses of plant roots to Pi starvation (Péret *et al.*, 2011). It was found here that overexpressing *PvSPX1* significantly inhibited hairy root growth at two P levels (Fig. 5B), but led to enlarged root hair zones (Fig. 6). This suggests that overexpression of *PvSPX1* could enhance root morphological modifications in adaptation to P deficiency.

In bean, it has been documented that the P-efficient genotype G19833 has greater root hair density and longer root hair length than the P-inefficient genotype DOR364 in low P conditions (Yan *et al.*, 2004). In this study, the Pi starvation-induced *PvSPX1* was originally cloned from G19833. Furthermore, higher *PvSPX1* expression levels were found in G19833 than in DOR364 (data not shown) at low P, suggesting positive contributions of *PvSPX1* to superior P efficiency in G19833 through regulation of root morphology. Subsequently, two genes regulating root growth in transgenic bean hairy roots, *PvLPR1-like* and *PvPDR2-like*, were cloned and their transcription was investigated Overexpression of *PvSPX1* led to increased expression of *PvLPR1-like* and reduced expression of *PvPDR2-like* (Fig. 7). Since it has been documented that *AtLPR1* and *AtPDR2* are two critical components regulating root growth in opposite ways (Ticconi *et al*., 2004, 2009; Reymond *et al.*, 2006; Svistoonoff *et al.*, 2007; Wang *et al.*, 2010; Miura *et al.*, 2011), it is conceivable that changes in root morphology in bean result from up-regulation of *PvSPX1*, with consequent effects on transcripts of *PvLPR1-like* and *PvPDR2-like*.

Although the three PvSPX proteins studied here exhibit high homology, and belong to the same subgroup in phylogenetic tree analysis (Fig. 1), diverse properties and functions of PvSPXs were observed in response to Pi starvation, as reflected by different expression patterns, variations in subcellular localization, and dissimilar growth of transgenic bean hairy roots. In response to Pi starvation, it seems that *PvSPX1* and *PvSPX2* might be earlier responsive genes which are more sensitive to Pi starvation than *PvSPX3* in bean leaves. At 4 d of Pi starvation, transcripts of both *PvSPX1* and *PvSPX2* in bean leaves were significantly increased, while *PvSPX3* remained unchanged (Fig. 2). Also, with an increase in available Pi from 100 μM to 500 μM in the medium, significantly decreased transcription was observed for *PvSPX1* and *PvSPX2*, but not for *PvSPX3* (Fig. 3). Interestingly, through their promoter-fused GUS activity analysis in bean hairy roots, it was found that *PvSPX1* and *PvSPX2* exhibited similar spatial expression patterns (Supplementary Fig. S7 at *JXB* online), and similar responses to P deficiency as well as subcellular localization (Figs 2, 4), but only overexpression of *PvSPX1* resulted in inhibited root growth, increased root P concentration, and changes of morphological traits in transgenic bean hairy roots (Figs 5, 6), strongly suggesting diverse functions of PvSPX members, and PvSPX requirement of other P signalling regulators to regulate P homeostasis and root growth in bean.

Similarly, diverse functions of *SPX* members have been demonstrated in *Arabidopsis* and rice (Duan *et al.*, 2008; C. Wang *et al.*, 2009). In *Arabidopsis*, suppressed *AtSPX3* led to an increased P concentration in shoots, and aggregative responses to Pi starvation (Duan *et al.*, 2008). However, knock-down of *AtSPX1*, *AtSPX2*, or *AtSPX4* did not alter the phenotypes of *Arabidopsis* at two P levels (Duan *et al.*, 2008). In rice, suppressed plant growth was observed through overexpression of *OsSPX1* and *OsSPX3*, as well as suppression of *OsSPX1* (C. Wang *et al.*, 2009; Z. Wang *et al.*, 2009). However, functions of other *SPX* members as related to P deficiency in rice still remain unknown.

**Fig. 9.**
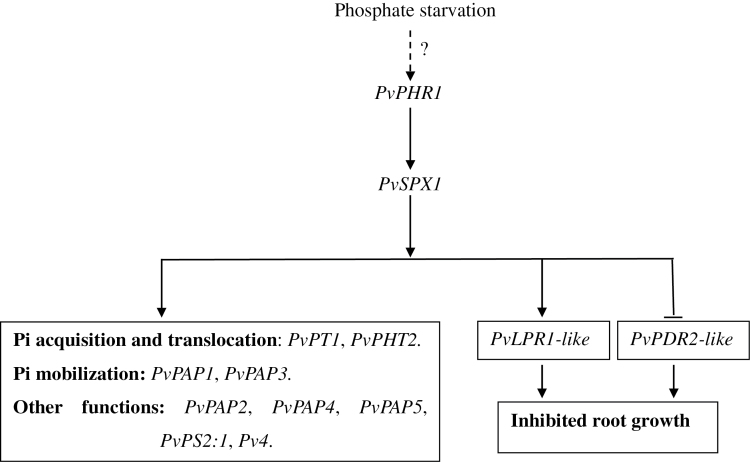
A suggested model for *PvSPX1* involvement in the P signalling network of bean. Arrowheads show the presence of positive regulation. Flat-ended lines show negative regulation. Dotted lines represent the putative regulatory pathway. The question mark indicates uncertainy in the network.

Taken together, the results demonstrate that PvSPX1 is a positive regulator in the P signalling network of common bean, and is downstream of PvPHR1 (Fig. 9). Increased transcription of *PvSPX1* led to significantly coordinated expressions of a group of Pi starvation-responsive genes, which could dramatically regulate changes of root morphology, Pi acquisition and mobilization, as well as P homeostasis in bean roots (Fig. 9).

## Supplementary data

Supplementary data are available at *JXB* online.

Figure S1. Expression of *PvSPX* genes in transgenic bean hairy roots.

Figure S2. Expression of *PvSPX1* in *PvSPX1* RNAi transgenic bean hairy roots.

Figure S3. Growth and P concentration of bean hairy roots in CK and *PvSPX1* RNAi transgenic lines at two P levels.

Figure S4. Percentage of root hair zone in bean hairy roots with suppressed *PvSPX1* at two P levels.

Figure S5. Transcription levels of downstream genes of *PvSPX1* in CK and *PvSPX1* RNAi transgenic lines.

Figure S6. Transcription levels of downstream genes of *PvSPX1* in CK and overexpression transgenic lines of *PvSPX2* or *PvSPX3*.

Figure S7. Expression patterns of *PvSPX1* and *PvSPX2* through their *promoter:GUS* analysis.

Table S1. List of primers used in the study.

Table S2. Effects of phosphorus availability on bean growth.

Supplementary Data

## References

[CIT0001] Battini JL Rasko JE Miller AD 1999 A human cell-surface receptor for xenotropic and polytropic murine leukemia viruses: possible role in G protein-coupled signal transduction. Proceedings of the National Academy of Sciences, USA 96, 1385–1390 10.1073/pnas.96.4.1385 PMC15472 9990033

[CIT0002] Bozzo GG Raghothama KG Plaxton WC 2002 Purification and characterization of two secreted purple acid phosphatase isozymes from phosphate-starved tomato (*Lycopersicon esculentum*) cell cultures. European Journal of Biochemistry 269, 6278–6286 12473124 10.1046/j.1432-1033.2002.03347.x

[CIT0003] Chiou TJ Lin S 2011 Signaling network in sensing phosphate availability in plants. Annual Review of Plant Biology 62, 185–206 10.1146/annurev-arplant-042110-103849 21370979

[CIT0004] Del Pozo JC Allona I Rubio V Leyva A De La Peña A Aragoncillo C Paz-Ares J 1999 A type 5 acid phosphatase gene from *Arabidopsis thaliana* is induced by phosphate starvation and by some other types of phosphate mobilising/oxidative stress conditions. The Plant Journal 19, 579–589 10504579 10.1046/j.1365-313x.1999.00562.x

[CIT0005] Duan K Yi K Dang L Huang H Wu W Wu P 2008 Characterization of a sub-family of Arabidopsis genes with the SPX domain reveals their diverse functions in plant tolerance to phosphorus starvation. The Plant Journal 54, 965–975 18315545 10.1111/j.1365-313X.2008.03460.x

[CIT0006] Fox TR Comerford NB 1990 Low-molecular-weight organic acids in selected forest soils of the southeastern USA. Soil Science Society of America Journal 54, 139–144

[CIT0007] Hamburger D Rezzonico E MacDonald-Comber PJ Somerville C Poirier Y 2002 Identification and characterization of the Arabidopsis *PHO1* gene involved in phosphate loading to the xylem. The Plant Cell 14, 889–902 11971143 10.1105/tpc.000745 PMC150690

[CIT0008] Hernández G Valdes-Lopez O Ramirez M 2009 Global changes in the transcript and metabolic profiles during symbiotic nitrogen fixation in phosphorus-stressed common bean plants. Plant Physiology 151, 1221–1238 19755543 10.1104/pp.109.143842 PMC2773089

[CIT0009] Kant S Peng M Rothstein SJ 2011 Genetic regulation by NLA and microRNA 827 for maintaining nitrate-dependent phosphate homeostasis in *Arabidopsis* . PLoS Genetics 7, e1002021 21455488 10.1371/journal.pgen.1002021 PMC3063762

[CIT0010] Lenburg ME O’Shea EK 1996 Signaling phosphate starvation. Trends in Biochemical Sciences 21, 383–387 8918192

[CIT0011] Liang C Chen Z Yao Z Tian J Liao H 2012a Characterization of two putative protein phosphatase genes and their involvement in phosphorus efficiency in *Phaseolus vulgaris* . Journal of Integrative Plant Biology 54, 400–411 22571280 10.1111/j.1744-7909.2012.01126.x

[CIT0012] Liang C Sun L Yao Z Liao H Tian J 2012b Comparative analysis of *PvPAP* gene family and their functions in response to phosphorus deficiency in common bean. PLoS One 7, e38106 22662274 10.1371/journal.pone.0038106 PMC3360649

[CIT0013] Liang C Tian J Lam HM Lim BL Yan X Liao H 2010 Biochemical and molecular characterization of PvPAP3, a novel purple acid phosphatase isolated from common bean enhancing extracellular ATP utilization. Plant Physiology 152, 854–865 19955264 10.1104/pp.109.147918 PMC2815866

[CIT0014] Liao H Yan X Rubio G Beebe SE Blair MW Lynch JP 2004 Genetic mapping of basal root gravitropism and phosphorus acquisition efficiency in common bean. Functional Plant Biology 31, 959–970 10.1071/FP03255 32688964

[CIT0015] Ligaba A Yamaguchi M Shen H Sasaki T Yamamoto Y Matsumoto H 2004 Phosphorus deficiency enhances plasma membrane H^+^-ATPase activity and citrate exudation in greater purple lupin (*Lupinus pilosus*). Functional Plant Biology 31, 1075–1083 10.1071/FP04091 32688975

[CIT0016] Lin SI Santi C Jobet E 2010 Complex regulation of two target genes encoding SPX-MFS proteins by rice miR827 in response to phosphate starvation. Plant and Cell Physiology 51, 2119–2131 21062869 10.1093/pcp/pcq170

[CIT0017] Liu F Wang Z Ren H Shen C Li Y Ling HQ Wu C Lian X Wu P 2010 OsSPX1 suppresses the function of OsPHR2 in the regulation of expression of *OsPT2* and phosphate homeostasis in shoots of rice. The Plant Journal 62, 508–517 20149131 10.1111/j.1365-313X.2010.04170.x

[CIT0018] Miura K Lee J Gong Q Ma S Jin JB Yoo CY Miura T Sato A Bohnert HJ Hasegawa PM 2011 *SIZ1* regulation of phosphate starvation-induced root architecture remodeling involves the control of auxin accumulation. Plant Physiology 155, 1000–1012 21156857 10.1104/pp.110.165191 PMC3032448

[CIT0019] Murphy J Riley J 1962 A modified single solution method for the determination of phosphate in natural water. Analytica Chimica Acta 27, 31–35

[CIT0020] Péret B Clément M Nussaume L Desnos T 2011 Root developmental adaptation to phosphate starvation: better safe than sorry. Trends in Plant Science 16, 442–450 21684794 10.1016/j.tplants.2011.05.006

[CIT0021] Raghothama KG 1999 Phosphate acquisition. Annual Review of Plant Physiology and Plant Molecular Biology 50, 665–693 10.1146/annurev.arplant.50.1.665 15012223

[CIT0022] Reymond M Svistoonoff S Loudet O Nussaume L Desnos T 2006 Identification of QTL controlling root growth response to phosphate starvation in *Arabidopsis thaliana* . Plant, Cell and Environment 29, 115–125 10.1111/j.1365-3040.2005.01405.x 17086758

[CIT0023] Robinson WD Park J Tran HT Del Vecchio HA Ying S Zins JL Patel K McKnight TD Plaxton WC 2012 The secreted purple acid phosphatase isozymes AtPAP12 and AtPAP26 play a pivotal role in extracellular phosphate-scavenging by *Arabidopsis thaliana* . Journal of Experimental Botany 63, 6531–6542 23125358 10.1093/jxb/ers309 PMC3504502

[CIT0024] Secco D Baumann A Poirier Y 2010 Characterization of the rice *PHO1* gene family reveals a key role for *OsPHO1;2* in phosphate homeostasis and the evolution of a distinct clade in dicotyledons. Plant Physiology 152, 1693–1704 20081045 10.1104/pp.109.149872 PMC2832267

[CIT0025] Secco D Wang C Arpat BA Wang ZY Whelan J 2012a The emerging importance of the SPX domain-containing proteins in phosphate homeostasis. New Phytologist 193, 842–851 22403821 10.1111/j.1469-8137.2011.04002.x

[CIT0026] Secco D Wang C Shou H Whelan J 2012b Phosphate homeostasis in the yeast *Saccharomyces cerevisiae*, the key role of the SPX domain-containing proteins. FEBS Letters 586, 289–295 22285489 10.1016/j.febslet.2012.01.036

[CIT0027] Spain BH Koo D Ramakrishnan M Dzudzor B Colicelli J 1995 Truncated forms of a novel yeast protein suppress the lethality of a G protein alpha subunit deficiency by interacting with the beta subunit. Journal of Biological Chemistry 270, 25435–25444 7592711 10.1074/jbc.270.43.25435

[CIT0028] Sparkes IA Runions J Kearns A Hawes C 2006 Rapid, transient expression of fluorescent fusion proteins in tobacco plants and generation of stably transformed plants. Nature Protocols 1, 2019–2025 10.1038/nprot.2006.286 17487191

[CIT0029] Stefanovic A Ribot C Rouached H Wang Y Chong J Belbahri L Delessert S Poirier Y 2007 Members of the *PHO1* gene family show limited functional redundancy in phosphate transfer to the shoot, and are regulated by phosphate deficiency via distinct pathways. The Plant Journal 50, 982–994 17461783 10.1111/j.1365-313X.2007.03108.x

[CIT0030] Ström L Owen AG Godbold DL Jones DL 2005 Organic acid behaviour in a calcareous soil implications for rhizosphere nutrient cycling. Soil Biology and Biochemistry 37, 2046–2054

[CIT0031] Svistoonoff S Creff A Reymond M Sigoillot-Claude C Ricaud L Blanchet A Nussaume L Desnos T 2007 Root tip contact with low-phosphate media reprograms plant root architecture. Nature Genetic 39, 792–796 10.1038/ng2041 17496893

[CIT0032] Taghipour M Jalali M 2012 Effect of low-molecular-weight organic acids on kinetics, release and fractionation of phosphorus in some calcareous soils of western Iran. Environmental Monitoring and Assessment 185, 5471–5482 23142876 10.1007/s10661-012-2960-y

[CIT0033] Tian J Venkatachalam P Liao H Yan X Raghothama K 2007 Molecular cloning and characterization of phosphorus starvation responsive genes in common bean (*Phaseolus vulgaris* L.). Planta 227, 151–165 17701202 10.1007/s00425-007-0603-2

[CIT0034] Tian J Wang X Tong Y Chen X Liao H 2012 Bioengineering and management for efficient phosphorus utilization in crops and pastures. Current Opinion in Biotechnology 23, 866–871 22445911 10.1016/j.copbio.2012.03.002

[CIT0035] Ticconi CA Delatorre CA Lahner B Salt DE Abel S 2004 *Arabidopsis pdr2* reveals a phosphate-sensitive checkpoint in root development. The Plant Journal 37, 801–814 14996215 10.1111/j.1365-313x.2004.02005.x

[CIT0036] Ticconi CA Lucero RD Sakhonwasee S Adamson AW Creff A Nussaume L Desnos T Abel S 2009 ER-resident proteins PDR2 and LPR1 mediate the developmental response of root meristems to phosphate availability. Proceedings of the National Academy of Sciences, USA 106, 14174–14179 10.1073/pnas.0901778106 PMC2723163 19666499

[CIT0037] Valdes-Lopez O Arenas-Huertero C Ramirez M Girard L Sanchez F Vance CP Luis RJ Hernández G 2008 Essential role of MYB transcription factor: PvPHR1 and microRNA: PvmiR399 in phosphorus-deficiency signalling in common bean roots. Plant, Cell and Environment 31, 1834–1843 10.1111/j.1365-3040.2008.01883.x 18771575

[CIT0038] Vance CP Uhde-Stone C Allan DL 2003 Phosphorus acquisition and use: critical adaptations by plants for securing a nonrenewable resource. New Phytologist 157, 423–447 10.1046/j.1469-8137.2003.00695.x 33873400

[CIT0039] Wang C Huang W Ying Y Li S Secco D Tyerman S Whelan J Shou H 2012 Functional characterization of the rice SPX-MFS family reveals a key role of *OsSPX-MFS1* in controlling phosphate homeostasis in leaves. New Phytologist 196, 139–148 22803610 10.1111/j.1469-8137.2012.04227.x

[CIT0040] Wang C Ying S Huang H Li K Wu P Shou H 2009 Involvement of *OsSPX1* in phosphate homeostasis in rice. The Plant Journal 57, 895–904 19000161 10.1111/j.1365-313X.2008.03734.x

[CIT0041] Wang LS Dong JS Gao ZY Liu D 2012 The Arabidopsis gene *HYPERSENSITIVE TO PHOSPHATE STARVATION 3* encodes ETHYLENE OVERPRODUCTION 1. Plant and Cell Physiology 56, 1093–1105 22623414 10.1093/pcp/pcs072

[CIT0042] Wang X Wang Y Tian J Lim BL Yan X Liao H 2009 Overexpressing *AtPAP15* enhances phosphorus efficiency in soybean. Plant Physiology 151, 233–240 19587103 10.1104/pp.109.138891 PMC2736008

[CIT0043] Wang Y Ribot C Rezzonico E Poirier Y 2004 Structure and expression profile of the Arabidopsis *PHO1* gene family indicates a broad role in inorganic phosphate homeostasis. Plant Physiology 135, 400–411 15122012 10.1104/pp.103.037945 PMC429393

[CIT0044] Wang Z Hu H Huang H Duan K Wu Z Wu P 2009 Regulation of OsSPX1 and OsSPX3 on expression of *OsSPX* domain genes and Pi-starvation signaling in rice. Journal of Integrative Plant Biology 51, 663–674 19566645 10.1111/j.1744-7909.2009.00834.x

[CIT0045] Yan X Liao H Beebe SE Blair MW Lynch JP 2004 QTL mapping of root hair and acid exudation traits and their relationship to phosphorus uptake in common bean. Plant and Soil 265, 17–29

[CIT0046] Yan X Liao H Trull MC Beebe SE Lynch JP 2001 Induction of a major leaf acid phosphates does not confer adaptation to low P availability in common bean. Plant Physiology 125, 1901–1911 11299369 10.1104/pp.125.4.1901 PMC88845

[CIT0047] Yang GZ Ding GD Shi L Cai HM Xu FS 2012 Characterization of phosphorus starvation-induced gene *BnSPX3* in *Brassica napus* . Plant and Soil 350, 339–351

[CIT0048] Zhou J Jiao F Wu Z Li Y Wang X He X Zhong W Wu P 2008 *OsPHR2* is involved in phosphate-starvation signaling and excessive phosphate accumulation in shoots of plants. Plant Physiology 146, 1673–1686 18263782 10.1104/pp.107.111443 PMC2287342

