# Supplementary material for: SPX1 is an important component in the phosphorus signalling network of common bean regulating root growth and phosphorus homeostasis
Source: J Exp Bot. 2014 Apr 30;65(12):3299–310. doi: 10.1093/jxb/eru183 (PMC4071846; doi:10.1093/jxb/eru183)
Supplement: Supplementary Data [file supp_65_12_3299__index.html]

 SPX1 is an important component in the phosphorus signalling network of common bean regulating root growth and phosphorus homeostasis — SPX1 is an important component in the phosphorus signalling network of common bean regulating root growth and phosphorus homeostasis — Supplementary Data 

# *SPX1* is an important component in the phosphorus signalling network of common bean regulating root growth and phosphorus homeostasis

## Supplementary Data

Data files

**Files in this Data Supplement:**

- Supplementary Data - Supplementary Data
