# Supplementary material for: SPX1 is an important component in the phosphorus signalling network of common bean regulating root growth and phosphorus homeostasis
Source: J Exp Bot. 2014 Apr 30;65(12):3299–310. doi: 10.1093/jxb/eru183 (PMC4071846; doi:10.1093/jxb/eru183)
Supplement: Supplementary Data [file supp_eru183_jexbot116061_file001.pdf]

Table S1 A list of primers used in the study

Gene	Forward primer (5'-3')	Reverse primer (5'-3')
T3	ATTAACCCTCACTAAAGGGA	
T7	TAATACGACTCACTATAGGG	
PvSPX2-5'	GGAAGAATGACGTTGAGA	
PvSPX3-5'	CAGCCCTTCTATGAAATTGA	
PvSPX3-3'	GCAGTGCTGATAAAGTTAGT	
PvSPX2C	ATGAAATTCGGAAAGAGCCTCA	CAGGTTACTTGGCTGCTTGTTTA
PvSPX3C	ACATGAAGTTTGAGAAGATCCT	AGCCTAGTTATGCTGAGAAG
OX-*PvSPX1*	GGATCCTATGAAATTCGG	ACGCGTTTACTTGGCTGTCTGTTCC
RNAi-PvSPX1(1)	GGATCCGACCTGCTCTACAAACTTGT	AAGCTTATCTGCAGCGGTGGC
RNAi-PvSPX1 (2)	CTGCAGATCTGCAGCGGTGG	ACGCGTGACCTGCTCTACAAACTTG
OX-*PvSPX2*	GGATCCTATGAAATTCGG	ACGCGTTTACTTGGCTGCTTGTT
OX-*PvSPX3*	GGATCCTATGAAGTTTGAGAAG	ACGCGTCTAGTTATGCTGAGAAG
OX-*PvPHR1*	GGATCCTATGTATCATTCAAAGAAT	ACGCGTTCACAGATTACTGC
EF-1α	TGAACCACCCTGGTCAGATT	TCCAGCATCACCATTCTTCA
PvSPX1-RT	GGCAACTCCCCAAGCTGAG	AAACCACCCATCTGCAGCG
PvSPX2-RT	TGAGATTGAGTACGTGGAGAGC	CAGGTTACTTGGCTGCTTGT
PvSPX3-RT	GCACCAGAGATAGCAACCT	CACACCCACCTACCATCACA
PvPDR2-RT	CTTCTGAATGCATTTTCTCCAACTC	GACGAATTATGTCAGTGGTAGGAGC
PvLPR1-RT	GTAACAACCAACGAGATCCTG	GGAATCCGTGACGTGTCA
Pv4-RT	GAATGTATAATAAGTTGAAAGATTGCT	ACACCCACATGAACACAACATGGC
PvPHT1-RT	GCAGTAACCTGGTTTTTGTTG	ACATCACAACAAACCCAATTCTG
PvPHT2-RT	GGAATAGTTGCAGGGCTAATG	CATCTACACTTGGATCTTGACTAGG
PvPHR1-RT	TACGGTGGACACATGAATTACAC	TCTGCACCTCCAATTGTTCA
PvSPX1GFP	TCTAGATATGAAATTCGGAAAGAGTC	GGATCCCGCTTGGCTGTCTGTTCCAG
PvSPX2GFP	TCTAGAGATGAAATTCGGAAAGAGCC	GGATCCAGCTTGGCTGCTTGTTT
PvSPX3GFP	TCTAGACATGAAGTTTGAGAAGATCC	GGATCCCGGTTATGCTGAGAAG
PvPAP1-RT	GCTTCCTTTGTCCTCTTGGT	TGTAACTGTGCCTTCTAAACTGG
PvPAP2-RT	GAGTAATGGAGGCAAAAGCAGCAAC	CATTGCCCTCCCCACAAGGTCAC
PvPAP3-RT	GAACGCTTGGTATTCCGTGT	TGTCTTCCCCAATCTCCAAC
PvPAP4-RT	GGTGTATGGTTGGCTTTCATTGGTG	GCAGAAACTTCAGATTGGTTGTAGG
PvPAP5-RT	GGAGGGGAGTTGTTGGTCAC	ACCCTGCTCCGCTAGTCAAG
PvPS2:1-RT	GCCCAAGTTTGAGGCTGATA	GAAGGGAGAATTGTGCTCCA

Table S2 Effects of phosphorus availability on bean growth

Days after Treatment (d)	Fresh weight (g. plant^{-1})				Total root length (m. plant^{-1})		Total P content (mg. plant^{-1})			
	Shoots		Roots				Shoots		Roots	
	-P	+P	-P	+P	-P	+P	-P	+P	-P	+P
4	16.58 (1.02)	18.67 (0.96)	5.38 (0.54)	7.41 (0.21)*	11.16 (0.93)	18.45 (1.67)*	2.02 (0.06)	2.43 (0.59)	27.43 (1.47)	43.07 (3.48)*
8	24.51 (1.03)	31.71 (1.22)*	10.51 (0.15)	15.33 (0.41)*	11.93 (0.38)	27.05 (2.56)*	2.63 (0.35)	3.89 (0.42)*	55.66 (4.58)	80.45 (1.83)*

Seedlings were hydroponically grown under normal conditions for 7 d, and then treated with Pi starvation. Shoots and roots were separately harvested for further analysis at indicated time. Data is the mean of four replicates with standard error in parenthesis. Asterisks represent significant differences of the same trait between two P treatments at 0.05 levels.

Fig. S1

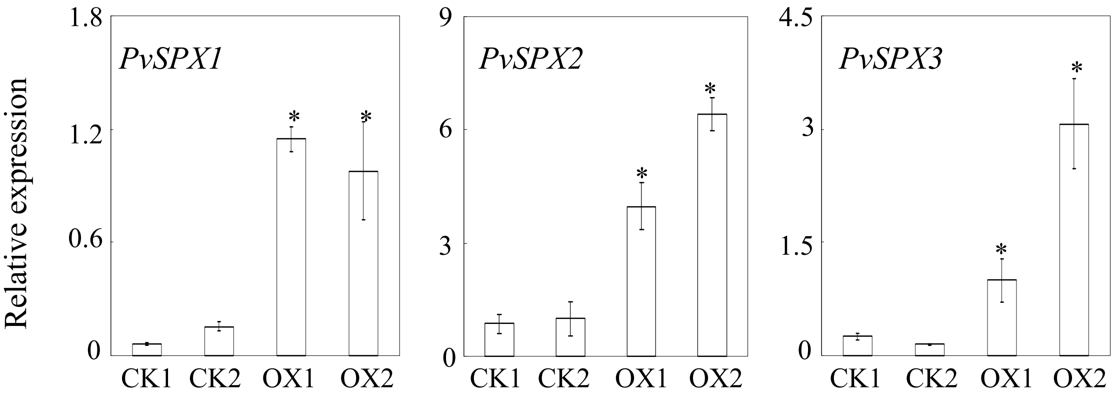

Fig. S1 Expression of *PvSPXs* in transgenic bean hairy roots. Expression levels of *PvSPXs* were determined in bean hairy roots grown in MS medium containing 1.25 mM phosphorus by qPCR. Expression levels of the tested genes were quantified relative to expression levels of the reference gene *EF-1α* (PvTC3216) using arbitrary units. OX1 and OX2 indicate two transgenic bean hairy root lines with overexpressing *PvSPX1*, *PvSPX2* or *PvSPX3*. CK1 and 2 indicate the two transgenic lines transformed with the empty vector. Asterisks indicate significant difference of *PvSPX* expression between overexpressing *PvSPX* and CK at 0.05 levels.

Fig. S2

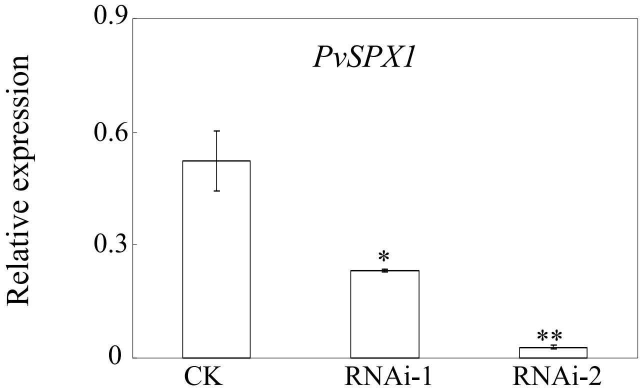

Fig. S2 Expression of *PvSPX1* in *PvSPX1* RNAi transgenic bean hairy roots. Expression levels of *PvSPX1* were determined in bean hairy roots grown in MS medium containing 1.25 mM phosphorus by qPCR. Expression levels of the tested genes were quantified relative to expression levels of the reference gene *EF-1α* (PvTC3216) using arbitrary units. RNAi-1/2: transgenic bean hairy roots with suppressed *PvSPX1* expression. CK: transgenic lines transformed with the empty vector. Asterisks indicate significant differences of *PvSPX1* expression in bean hairy root between RNAi and CK. *: $0.01<p\leq0.05$; **: $p\leq0.01$.

Fig. S3

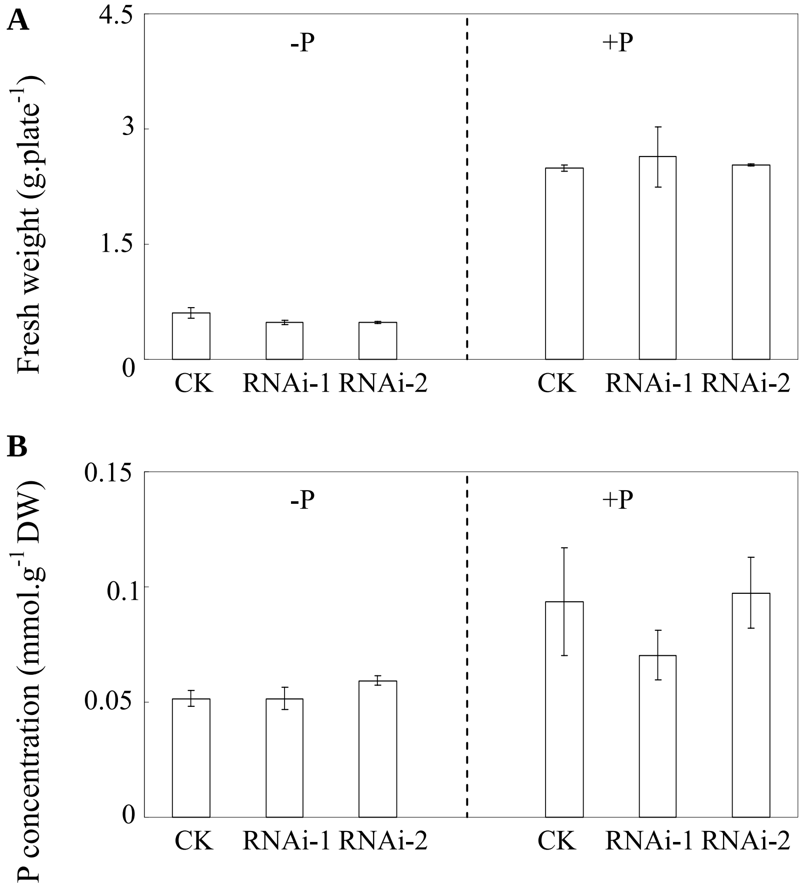

Fig. S3 Growth and P concentration of bean hairy roots in CK and *PvSPX1* RNAi transgenic lines at two P levels. A) Fresh weight of bean hairy roots at two P levels; B) Phosphorus concentration in bean hairy roots. Bean hairy roots were grown in the medium containing 0 µM (-P) or 1.25 mM (+P) KH_2PO_4 for 14 d. Fresh weight and P concentration was separately measured. Each bar is the mean of four replicates with standard error. RNAi-1/2: transgenic lines with suppressing *PvSPX1*. CK: transgenic line transformed with the empty vector.

Fig. S4

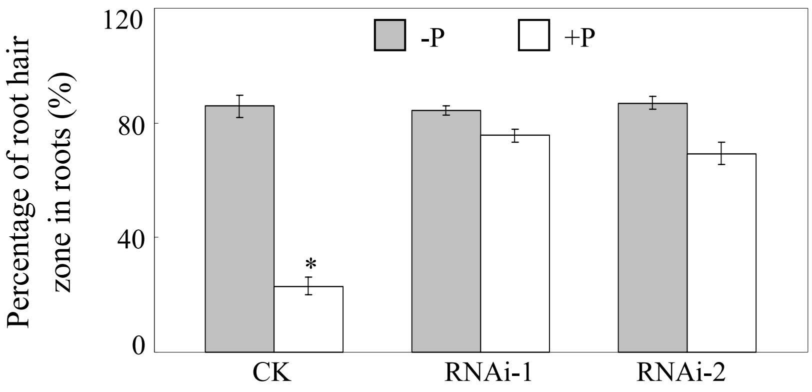

Fig. S4 Percentage of root hair zone in bean hairy roots with suppressed *PvSPX1* at two P levels. Bean hairy roots were grown in the medium containing 0 µM (-P) or 1.25 mM (+P) KH_2PO_4 for 14 d. Ten lateral roots were selected to measure root length in different regions from each replicate. RNAi-1/2: two transgenic lines with suppressing *PvSPX1*. CK: transgenic line transformed with the empty vector. Asterisks represent significant differences between two P treatments at 0.05 levels.

Fig. S5

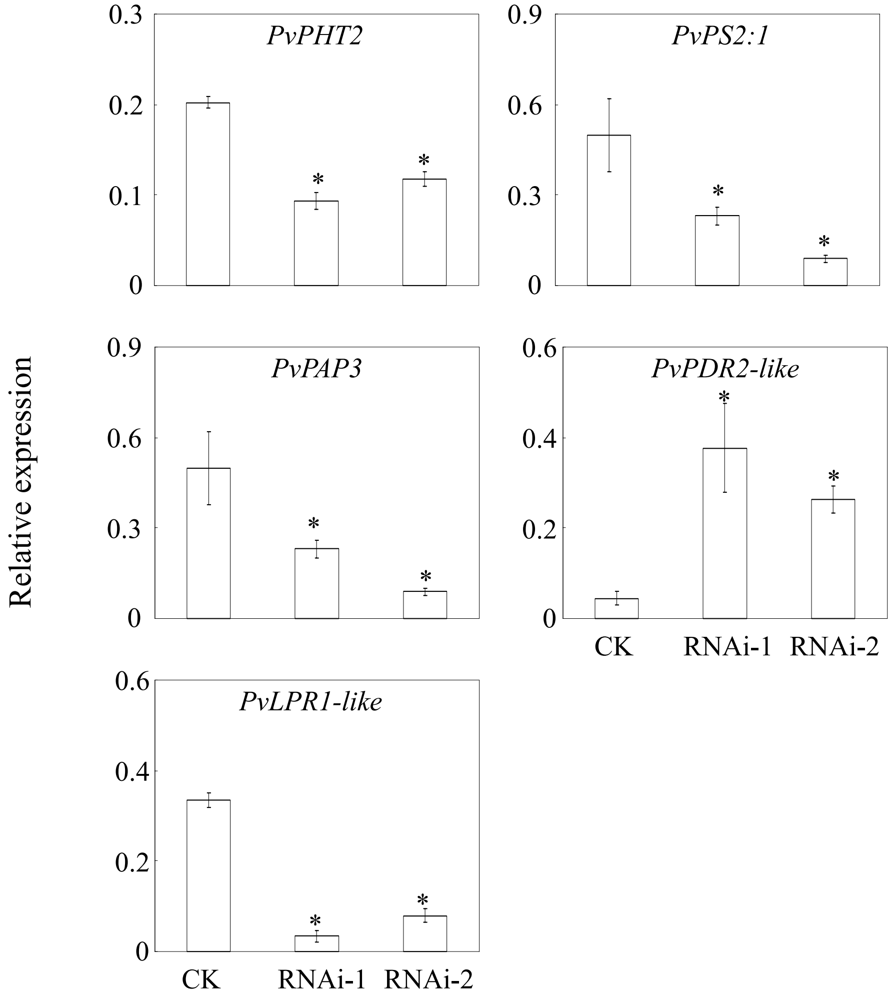

Fig. S5 Transcription levels of down stream genes of *PvSPX1* in CK and *PvSPX1* RNAi transgenic lines. Expression patterns of down stream genes were separately determined in CK and two *PvSPX1* RNAi lines grown in the MS medium containing 1.25 mM phosphorus. Expression levels of the tested genes were quantified relative to expression levels of the reference gene *EF-1α* (PvTC3216) using arbitrary units. RNAi-1/2: two transgenic bean hairy root lines with suppressing *PvSPX1*. CK: transgenic line transformed with the empty vector. Asterisks represent significant differences of gene transcripts between *PvSPX1* RNAi and CK lines at 0.05 levels.

Fig. S6

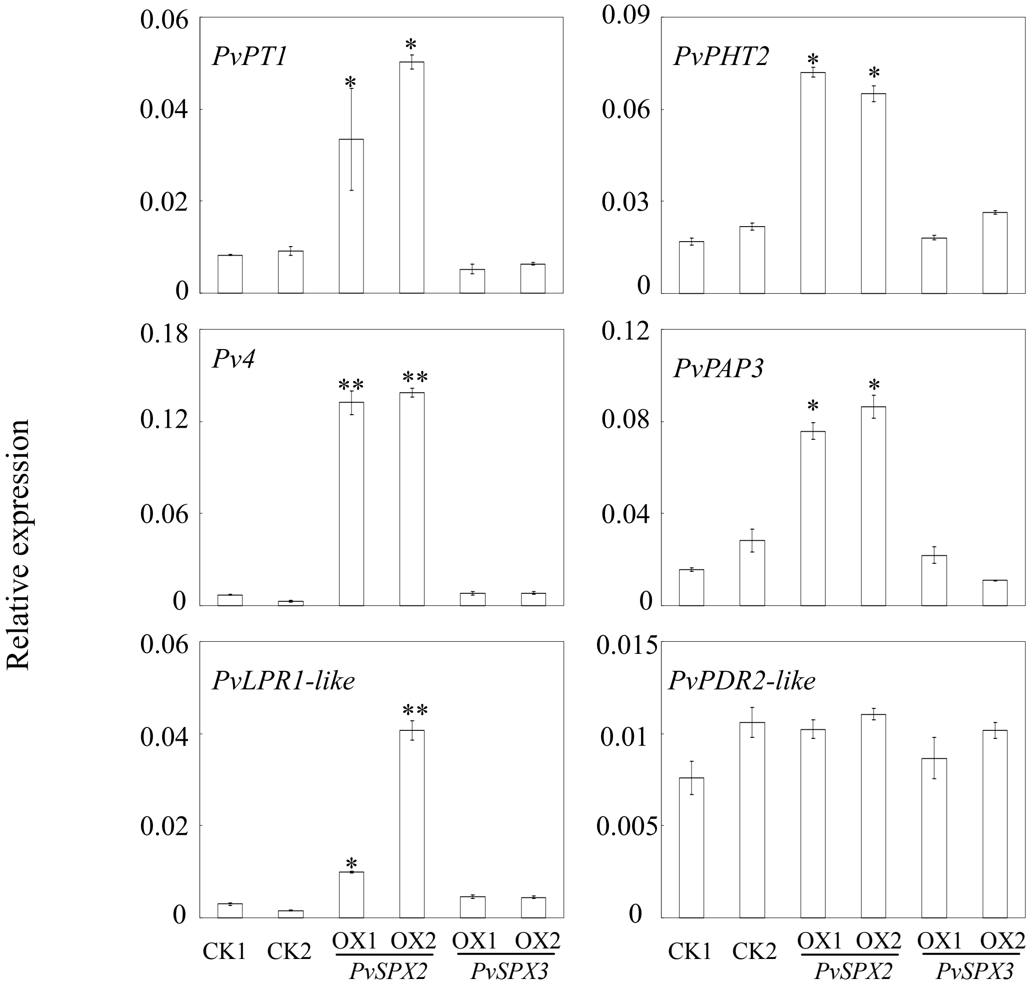

Fig. S6 Transcription levels of down stream genes of *PvSPX1* in CK and overexpression transgenic lines of *PvSPX2* or *PvSPX3*. Expression patterns of down stream genes were separately determined in CK and two *PvSPX2* or *PvSPX3* overexpression lines grown in the MS medium containing 1.25 mM phosphorus. Expression levels of the tested genes were quantified relative to expression levels of the reference gene *EF-1α* (PvTC3216) using arbitrary units. OX1 and OX2 indicate two transgenic bean hairy root lines with overexpressing *PvSPX2* or *PvSPX3*. CK1 and CK2 indicate the two transgenic lines transformed with the empty vector. Asterisks represent significant differences of gene transcripts between CK and *PvSPX2* or *PvSPX3* overexpression lines. *: $0.01<p\leq0.05$; **: $p\leq0.01$.

Fig. S7

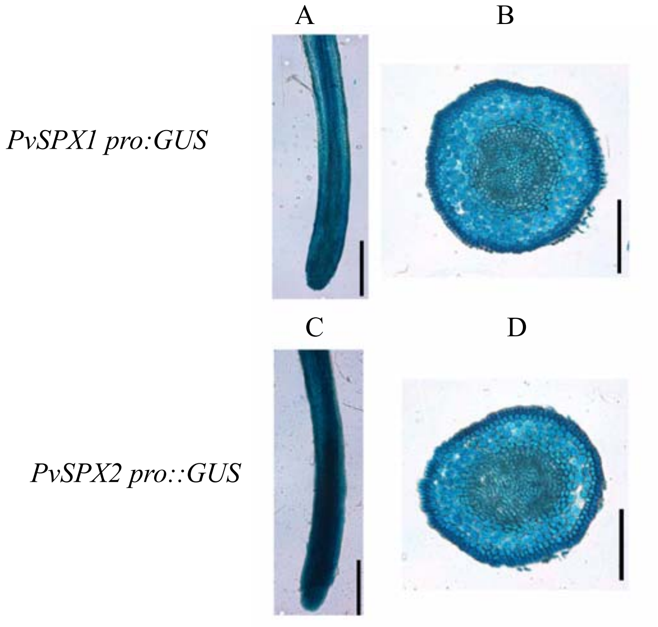

Fig. S7 Expression patterns of *PvSPX1* and *PvSPX2* through their *promoter:GUS* analysis. A and B: Longitudinal section (A) and cross section (B) of bean hairy roots transformed with *PvSPX1 Pro:GUS*; C and D: Longitudinal section (C) and cross section (D) of bean hairy roots transformed with *PvSPX2 Pro::GUS*. Scale bar is 0.5 cm for A and C, 0.2 cm for B and D.
